# Quantifying mammalian genomic DNA hydroxymethylcytosine content using solid-state nanopores

**DOI:** 10.1038/srep29565

**Published:** 2016-07-07

**Authors:** Osama K. Zahid, Boxuan Simen Zhao, Chuan He, Adam R. Hall

**Affiliations:** 1 Virginia Tech-Wake Forest University School of Biomedical Engineering and Sciences, Wake Forest University School of Medicine, Winston-Salem, NC 27101, USA; 2 Department of Chemistry, Department of Biochemistry and Molecule Biology, Institute for Biophysical Dynamics, The University of Chicago, 929 East 57th Street, Chicago, Illinois 60637, USA; 3 Howard Hughes Medical Institute, The University of Chicago, 929 East 57th Street, Chicago, Illinois 60637, USA; 4 Comprehensive Cancer Center, Wake Forest University School of Medicine, Winston-Salem, NC 27101, USA

## Abstract

5-hydroxymethylcytosine (5 hmC), the oxidized form of 5-methylcytosine (5 mC), is a base modification with emerging importance in biology and disease. However, like most epigenetic elements, it is transparent to many conventional genetic techniques and is thus challenging to probe. Here, we report a rapid solid-state nanopore assay that is capable of resolving 5 hmC with high specificity and sensitivity and demonstrate its utility in assessing global modification abundance in genomic DNA.

Since its positive identification in the mammalian genome in 2009^1^,^2^, the epigenetic DNA modification 5 hmC has been the subject of intense investigation. Formed through the action of the ten eleven translocation (TET) family of dioxygenases, 5 hmC acts both as an intermediate in the active demethylation pathway and as an independent regulatory element, playing an important role in cell differentiation^3^, development^4^,^5^,^6^, aging and neurological disorders^7^,^8^. While positioning relative to the genetic sequence is critical^5^, global levels of this modification have also been shown to be an important biomarker. For example, overall 5 hmC abundance is significantly depleted across diverse cancers in comparison to healthy tissue^9^. As a result, quantification of 5 hmC levels in cell-free DNA, for instance, could be a valuable tool for minimally-invasive monitoring of cancer initiation and progression. However, current methods for probing 5 hmC either lack sensitivity or are too expensive or time-consuming to implement for regular clinical use, making the development of alternative approaches essential.

The single-molecule approach of solid-state (SS-)nanopores^10^ is an important candidate to address this challenge, with precision and sensitivity that make it attractive for an array of applications, including analytical devices^11^ and next-generation sequencers^12^. In the general approach, a nanometer scale aperture separates two chambers filled with electrolyte solution and a voltage applied between the chambers generates an ionic current (Fig. 1a). The electrically-facilitated threading of molecules through the nanopore is marked by a transient change in this current (an “event”), through which properties of the translocating material can be obtained. We have developed^13^ a SS-nanopore assay that produces significant events only when short, double-stranded^14^ DNA (dsDNA) binds to a chaperone protein (monovalent streptavidin, or MS^15^). Analyzed individually with a SS-nanopore of ~8 nm diameter, neither MS nor dsDNA generate significant events (c.f. Supplementary Fig. S1). The MS is both small and highly charged (−17.1*e*), owing to a covalent hexaglutamate tag used for isolation^15^, and therefore passes through the pore at speeds beyond the resolution limits of conventional electronics^16^. While a few reports have demonstrated detection of short nucleic acids^17^,^18^,^19^,^20^, these have all been conducted using either SS-nanopores fabricated specifically to do so^17^,^18^,^19^, in ultra-thin membranes or with very small diameters, or using high bandwidth electronics to increase temporal resolution^20^. However, in more conventional systems, such molecules also translocate too rapidly to be detected. In contrast, the bulkier nucleoprotein complex interacts sterically with the SS-nanopore walls as it passes, resulting in prolonged, resolvable events. This intrinsic selectivity enables detection and quantification of dsDNA molecules containing a biotin moiety. We recently applied an variation of this assay to the detection of specific nucleic acid sequences, including microRNAs^14^. Here, we extend the technique to probe 5 hmC epigenetic modifications in genomic DNA.

## Results

### Assay demonstration with synthetic oligonucleotides

**Figure 1:**
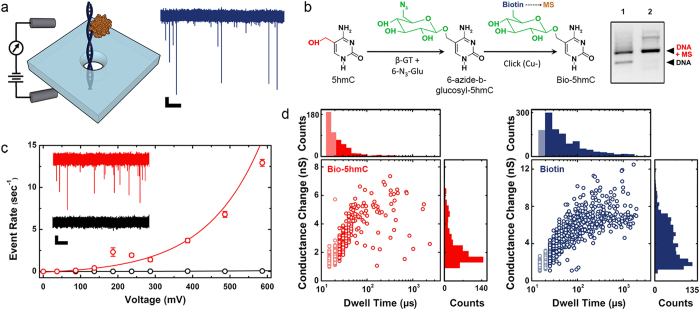
Nanopore detection of 5 hmC modification (**a**) Schematic of measurement setup depicting a short biotinylated dsDNA bound by MS translocating through a SS-nanopore (left) and an example current trace for synthetic monobiotinylated 156 bp dsDNA + MS measured at 400 mV (right). Scale bars are 1 nS (vertical) and 250 ms (horizontal). (**b**) 5 hmC labeling scheme, consisting of glucosylation by βGT with 6-N_3_-Glu to form 6-azide-b-glucosyl-5 hmC and biotin functionalization by copper-free click chemistry. Right: EMSA showing biotin-labeled mono-5 hmC (lane 1) and synthetic monobiotinylated dsDNA (lane 2), both with excess MS. (**c**) Event rate vs. applied voltage for 156 bp mono-5 hmC dsDNA (550 nM) biotin-labeled at 5 hmC sites both with (red) and without (black) bound MS measured through a pore with diameter 8.5 nm. Solid lines are exponential fits to the data. Inset shows example current traces from each (400 mV, colors matched). Scale bars are 1 nS and 250 ms. (**d**) Scatter plots of mean event dwell time vs. mean conductance change collected and accompanying histograms for biotin-labeled mono-5 hmC (red, diameter 8.5 nm) and synthetic monobiotinylated dsDNA (blue, diameter 7.8 nm) measured at 400 mV. Faded regions represent events below the resolution limit.

Because our assay is selective for biotinylated DNA, arbitrary base modifications can in principle be targeted for quantification through specific addition of a biotin linker to their structures. To study 5 hmC, we utilize a strategy pioneered by Song *et al*.^5^ that utilizes the T4 bacteriophage enzyme β-glycosyltransferase (β-GT) to transfer a glucose moiety specifically to 5 hmC (Fig. 1b). Through the use of a synthetic UDP-6 deoxy-6-azido-α-D-glucopyranoside (6-N_3_-Glu), the incorporated azide group can subsequently be used for selective biotin functionalization through copper-free click chemistry. As an initial demonstration, we first employ the method to biotinylate a synthetic 156 bp dsDNA containing a single 5 hmC nucleotide near its end (Supplementary Table 1), confirmed subsequently by electromobility shift assay (EMSA, Fig. 1b, right). SS-nanopore investigation of this material yields an increase in capture rate of more than an order of magnitude compared to an unlabeled control (Fig. 1c). This observation is comparable to data collected with 156 bp dsDNA synthesized with a biotin at the same position (Supplementary Figs 2–4). We attribute the minor quantitative disparity to a difference in pore diameter (Supplementary Table 2). Collectively, these results demonstrate two crucial points about the assay: first, the biotin label residing near the oligonucleotide end has the same effect as a center label^13^, showing that modification position is not a major factor; and second, the bulky (605 Da) glucose linker used here does not significantly affect detection viability, showing the adaptability of the assay.

### Determining hmC content in murine genomic DNA

**Figure 2:**
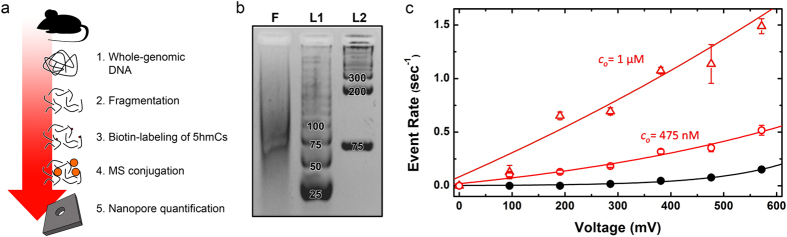
SS-nanopore detection of 5 hmC in mouse genomic DNA (**a**) Scheme showing preparation of genomic DNA for the SS-nanopore assay. (**b**) Gel electrophoresis of fragmented mouse genomic DNA (‘F’) and two low-molecular weight DNA ladders (L1 and L2, band labels are in bp). (**c**) Event rate vs. applied voltage measured for genomic DNA treated as described in (**a**) at total DNA concentrations *c*_*o*_ of 1 μM (red triangles) and 475 nM (red circles) in the presence of excess MS with 9.1 and 9.3 nm diameter nanopores, respectively. Black data points are a negative control of the same material (no MS). Solid lines are exponential fits to the data.

Having established an ability to assess 5 hmC specifically with SS-nanopores, we next apply our technique to quantify modification levels in mammalian DNA using a methodology that is shown schematically in Fig. 2a. For this investigation, we focus on genomic DNA extracted from murine brain tissue because of the known abundance of 5 hmC in functional elements of the brain^1^ and its suggested role in associated disorders^8^,^21^. The selectivity of our SS-nanopore approach requires short dsDNA because long molecules produce signals without MS binding^10^. Therefore, prior to assessment, the length of isolated whole genomic DNA must be reduced. For this, we utilize focused ultra-sonication to fragment the DNA to an average length of 75 bp (Fig. 2b). We note that the variation in fragment length is approximately symmetric with respect to the mean; since the capture rate for our assay varies linearly with dsDNA length^22^ from roughly 50 to 250 bp (Supplementary Fig. 5), SS-nanopore analysis is therefore representative of the mean substrate population. Fragmented DNA is subsequently subjected to the 5 hmC biotinylation process, purified, and measured by SS-nanopore. We again observe a pronounced event rate enhancement for the labeled material in the presence of MS at two total DNA concentrations *c*_*o*_ (1 μM and 475 nM, Fig. 2c) compared to the rate for a control measurement of fragmented DNA without MS.

**Figure 3:**
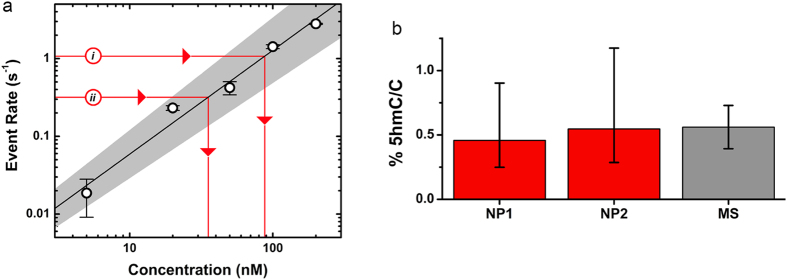
Quantification of 5 hmC content by SS-Nanopore assay (**a**) Event rate vs. concentration (400 mV) for 75 bp monobiotinylated dsDNA with excess MS. Solid line is a linear fit to the data and shaded region represents error of fit. Solid red lines show experimentally-derived event rate values for mouse genomic DNA, biotin-labeled at 5 hmC sites, and in the presence of excess MS, for *i*: NP1 (*c*_*o*_ = 1 μM, diameter 9.1 nm) and *ii*: NP2 (*c*_*o*_ = 475 nM, diameter 9.3 nm). (**b**) Quantification of 5 hmC/C ratio *R* determined for the values in (**a**) and comparison with LC-MS/MS measurements of the same material. Error bars for nanopore measurements represent error of the fit from (**a**).

To validate the measurement, we finally compare our results to quantification by the established technique of liquid chromatography-tandem mass spectrometry (LC-MS/MS). LC-MS/MS is a widely used and exquisitely sensitive analytical procedure that can quantify 5 hmC relative to unmodified cytosines (C)^23^. However, it is time consuming and relies on expensive and complex instrumentation, and so achieving similar results with a rapid, low cost system would be a tremendous advance. To accomplish this with our SS-nanopore assay, we implement here a simple expression for the 5 hmC/C ratio, *R*:


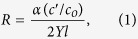


where *α* is the fractional cytosine content of the genome, *c*′ is the concentration of biotinylated fragments, *Y* is the biotin labeling yield, and *l* is the mean dsDNA fragment length in bp. For our experiments, we take *α* to be 0.21 based on the G+C content of the mouse genome^24^ and *l* as 75 bp (c.f. Fig. 2b). The total input DNA concentration, *c*_*o*_, is determined by spectrophotometry. The yield, *Y*, for the labeling methodology has been reported^5^ as high as 90%, but can vary based on experimental factors and therefore must be determined for each sample. For precision, we use here the LC-MS/MS analysis of labeled and unlabeled DNA to find *Y* of 51% for our material. However, this variable can also be determined through less demanding approaches, including EMSA with simultaneously labeled synthetic oligonucleotides. Finally, the labeled fragment concentration, *c*′, can be determined through comparison of experimental SS-nanopore event rate data to a standard curve generated by measuring the rate of synthetic 75 bp monobiotinylated dsDNA through a similar pore (Fig. 3a). Figure 3b shows 5 hmC/C quantification results from the two independent measurements of different total input DNA concentrations shown in Fig. 2c. For the first devce (NP1, *c*_*o*_ = 1 μM), we find an *R* of 0.46 (+0.45, −0.21)%, while the second device (NP2, *c*_*o*_ = 475 nM) yields an *R* of 0.55 (+0.63, −0.26)%. These values compare remarkably well to the ratio determined by LC-MS/MS of 0.56 ± 0.17%. The error in the SS-nanopore quantifications is taken as the error of the fit to the standard curve in Fig. 3a. This is a conservative estimation that we consider larger than the uncertainty in each of the variables from which *R* is derived. The error can be reduced with longer or additional measurements of synthetic standards to minimize uncertainty. We also note that our expression does not strictly quantify 5 hmC relative to unmodified C, like LC-MS/MS, but rather to total (modified and unmodified) C content. However, the low cumulative levels of cytosine modifications, predominantly^6^ 5mC, in mouse brain DNA would result in a difference of <2% in *R*; a value that is within the error of our measurements.

Our analysis assumes only that 5 hmC is sparse- an argument that is supported generally by its established low abundance in genomic DNA (<1% in both mouse^25^ and human^26^ tissues). As a result, we expect that labeled DNA fragments will tend to feature either one or zero biotins. While CpG dinucleotides are known to often reside in close proximity to one another, forming “islands”^27^ that may result in multiple 5 hmC on a single fragment and potential undercounting, the characteristics of labeled genomic fragment events are similar to those of synthetic monobiotinylated 75 bp dsDNA (Supplementary Fig. 6). This suggests that their respective conformations are comparable and the quantitative agreement with LC-MS/MS further supports the validity of our assumption.

## Discussion

SS-nanopores have been employed to detect epigenetic DNA modifications previously, through monitoring changes in translocation dynamics associated with modification-induced DNA structural differences^28^,^29^ and through steric labeling of 5 mC with methyl-binding proteins^30^. However, the minimum abundance of 5 hmC necessary to produce resolvable structural variation in the former case (~3%) is considerably higher than physiological abundance and there are currently no known 5 hmC-binding proteins for measurements analogous to the latter case. Additionally, neither previous approach is intrinsically selective, thus requiring significant analysis efforts and meticulous interpretation of the electrical signal for differentiation.

In response, we have combined a new SS-nanopore approach for detecting biotinylated oligonucleotides with an enzymatic technique for addition of a biotin linker to 5 hmC sites, resulting in a novel assay capable of detecting and quantifying 5 hmC content in DNA. First, we established the measurement with a synthetic dsDNA substrate (156 bp), demonstrating the detection of molecules containing a single 5 hmC. We then used the approach to assess native modification abundance in genomic DNA derived from murine brain tissue. Comparison with the established technique of LC-MS/MS showed comparable quantification sensitivity. To our knowledge, this work represents the first measurement of a physiologically-relevant factor in mammalian genomic DNA using SS-nanopores. These results demonstrate the value of the platform in probing 5 hmC modifications with speed and precision, suggesting its possible future use in disease diagnostics and clinical screening. We anticipate that our approach can also be expanded to other modifications using alternative biotin-labeling strategies^31^,^32^, offering potential modularity and targeting of additional biomarkers types.

## Methods

### Biomolecule preparation

Synthetic dsDNA oligonucleotides (156 bp or 75 bp) containing either a single 5 hmC or biotin modification were prepared by PCR using a modified primer (Supplementary Table 1) and genomic bacteriophage Lambda DNA (New England BioLabs, Ipswich, MA) as a template. Following the PCR reaction, dsDNA products were purified using a QIAquick purification kit (Qiagen, Inc., Valencia, CA) and eluted in pure deionized water (EMD Millipore, Billerica, MA). PCR products were quantified with a NanoDrop 2000 spectrophotometer (Thermo Fisher Scientific, Waltham, MA). All synthetic oligonucleotides were obtained commercially (Integrated DNA Technologies, Coralville, IA), suspended in deionized water to a stock concentration of 200 μM, and stored at −20 °C prior to use. All PCR and annealing products were confirmed by gel electrophoresis, wherein samples were loaded on a 2.5% agarose gel prepared in 1X TBE with GelRed nucleic acid stain (Phenix Research Products, Candler, NC) and imaged using a Gel Doc^TM^ system (BioRad, Hercules, CA).

### 5 hmC labeling reaction

5 hmC sites were labeled following the protocol developed by Song *et al*.^5^. The labeling reaction was performed at total concentrations of 50 mM HEPES buffer (pH 7.9), 25 mM MgCl_2_, 1 mM DDT, 250 μM UDP-azide-gluocose, 0.5 μM of T4-βGT, and 100 ng/μL DNA (either synthetic hmC oligonucleotides or fragmented mouse genomic DNA) and incubated at 37 °C for 2 hours. Subsequently, 350 μM sulfo-dibenzocyclooctyne-biotin was added to the solution and incubated at 37 °C overnight. The labeled DNA samples were then purified using a QIAquick purification kit and eluted in pure deionized water. Reaction components were taken from the Hydroxymethyl Collector^TM^ Kit (Active Motif, Carlsbad, CA) except T4-βGT, purchased from New England BioLabs, and sulfo-dibenzocyclooctyne-biotin purchased from Sigma-Aldrich (St. Louis, MO).

### Mouse genomic DNA preparation

Mouse genomic DNA (1 μg/μL) extracted from brain tissue was obtained commercially (Zyagen, San Diego, CA) and stored at −20 °C prior to use. Sample fragmentation was performed by suspending 10 μg of genomic DNA in pure deionized water to a final volume of 50 μL in a microTUBE AFA fiber snap-cap (Covaris, Woburn, MA) followed by shearing with a Covaris S220 focused-ultrasonicator operated at the following settings: peak incident power = 175 W; duty factor = 10%; cycles per burst = 200; total treatment time = 280 s. Fragment length was confirmed by gel electrophoresis on 4% agarose gel as described above. For control material, fragmented genomic DNA was purified immediately using a QIAquick purification kit (Qiagen, Valencia, CA), eluted in pure deionized water, and stored at −20 °C prior to nanopore measurement.

### Electromobility shift assay (EMSA)

Synthetic monobiotinylated or biotin-labeled mono-5 hmC dsDNA (156 bp, 0.3 μM) was incubated in 1X PBS with 1.5 μM MS for 10 min at room temperature. Samples were subsequently loaded on a 2.5% agarose gel with GelRed nucleic acid stain for visualization and run in an ice bath to prevent excessive diffusion of bands during migration. Imaging was performed as described above.

### Binding reaction incubation

MS^15^, a 54.5 kDa mutant streptavidin variant (SAe1D3) containing a single biotin-binding site, was supplied by the Howarth lab (Oxford University). For all experiments, 2.5 μM MS was incubated at molar excess with DNA target in 1X PBS buffer for 10–15 min at room temperature. After incubation the mixture was brought to a final salt concentration of 900 mM NaCl and 0.5X PBS.

### Nanopore fabrication, detection and analysis

4.4 mm silicon chips containing a 25 nm thin free-standing SiN membrane were obtained commercially (Norcada, Inc., Alberta, Canada). In each membrane, an individual nanopore 7.8–9.5 nm in diameter was fabricated with an Orion Plus helium ion microscope (Carl Zeiss, Peabody, MA) using a technique described elsewhere^33^. Following fabrication, chips were stored in 50% ethanol solution until use. Before measurement, a chip was rinsed with deionized water and ethanol, and dried by filtered air. After exposure by air plasma (30 W) for 2 min on each side, the chip was placed in a custom Ultem 1000 flow cell and measurement buffer was added immediately to both sides. Voltage was applied and current measured through Ag/AgCl electrodes using an Axopatch 200B patch clamp amplifier (Molecular Devices, Sunnyvale, CA). Pores were verified by measuring linear IV characteristics with a steady baseline and resistance commensurate with intended diameter. Current traces were collected at a rate of 200 kHz with a 100 kHz four-pole Bessel filter and analyzed with custom software, with which an additional 25 kHz low-pass filter was applied to all data. An event was defined as having amplitude above a threshold of 4.5σ and duration between 12.5 to 2400 μs. Below the temporal resolution limit (c.f. Fig. 1d), event metrics may be skewed but can still be identified as events. Rate was determined by measuring uninterrupted current traces of identical time durations for each data set (See Supplementary Table 2 for details). Data was saved in increments of 3.2 s and the standard deviation of the rates was used as measurement error.

### Liquid chromatography-tandem mass spectrometry

DNA was boiled at 95 °C for 5 min and digested by 1 U nuclease P1 (Wako Chemicals, Richmond, VA) in 25 μl of buffer containing 10 mM NH_4_OAc (pH 5.3) at 42 °C overnight. Next, NH_4_HCO_3_ (1 M, 3 μl) and 0.001 U venom phosphodiesterase (Sigma) were added and incubated at 37 °C for 2 h before a final treatment with 0.5 U alkaline phosphatase (Sigma) at 37 °C for 2 h. After the digestion steps, samples were diluted to 50 μl and filtered (0.22 μm pore size, 4 mm diameter; EMD Millipore), and 10 μl of the solution was injected into LC-MS/MS for analysis. Nucleosides were separated by reverse phase ultra-performance liquid chromatography on a C18 column (#927700–902; Agilent Technologies, Santa Clara, CA) at 35 °C with on-line mass spectrometry detection using an Agilent 6410 QQQ triple-quadrupole LC mass spectrometer in positive electrospray ionization mode. Soluble phase A was dionized water with 0.1% formic acid and phase B was methanol with 0.1% formic acid. The nucleosides were quantified by using the nucleoside to base ion mass transitions of 228 to 112 (C) and 258 to 142 (5 hmC). Quantification was performed by comparison to a curve obtained with pure nucleoside standards running on the same batch of samples. The 5 hmC/C ratio was calculated based on the calibrated concentrations.

## Additional Information

**How to cite this article**: Zahid, O. K. *et al*. Quantifying mammalian genomic DNA hydroxymethylcytosine content using solid-state nanopores. *Sci. Rep.* **6**, 29565; doi: 10.1038/srep29565 (2016).

## Supplementary Material

Supplementary Information
