# Author Correction: Quantifying mammalian genomic DNA hydroxymethylcytosine content using solid-state nanopores

**DOI:** 10.1038/s41598-024-58474-0

**Published:** 2024-04-03

**Authors:** Osama K. Zahid, Boxuan Simen Zhao, Chuan He, Adam R. Hall

**Affiliations:** 1 Virginia Tech-Wake Forest University School of Biomedical Engineering and Sciences, Wake Forest University School of Medicine, Winston-Salem, NC 27101 USA; 2 Department of Chemistry, Department of Biochemistry and Molecule Biology, Institute for Biophysical Dynamics, The University of Chicago, 929 East 57th Street, Chicago, IL 60637 USA; 3 Howard Hughes Medical Institute, The University of Chicago, 929 East 57th Street, Chicago, IL 60637 USA; 4 Comprehensive Cancer Center, Wake Forest University School of Medicine, Winston-Salem, NC 27101 USA

Correction to: *Scientific Reports* 10.1038/srep29565, published online 07 July 2016

This Article contains an error in the equation for estimating the 5 hmC/C ratio, *R*.

In the Results section, “Determining hmC content in murine genomic DNA”

$$ R = \frac{{\alpha \left( {{\raise0.7ex\hbox{${c^{\prime } }$} \!\mathord{\left/ {\vphantom {{c^{\prime } } {c_{o} }}}\right.\kern-\nulldelimiterspace} \!\lower0.7ex\hbox{${c_{o} }$}}} \right)}}{{2lY}} $$

should read:

$$R=\frac{\left(\frac{{c}{\prime}}{{c}_{o}}\right)}{2lY\mathrm{\alpha }}$$

