# Supplementary material for: Quantifying mammalian genomic DNA hydroxymethylcytosine content using solid-state nanopores
Source: Sci Rep. 2016 Jul 7;6:29565. doi: 10.1038/srep29565 (PMC4935868; doi:10.1038/srep29565)
Supplement: Supplementary Information [file srep29565-s1.pdf]

Supplementary Information

Quantifying mammalian genomic DNA hydroxymethylcytosine content using solid-state nanopores

Osama K. Zahid, Boxuan Simen Zhao, Chuan He, and Adam R. Hall

Oligonucleotide	Sequence (5'→ 3')
Biotinylated Reverse Primer	CAGTTGAGGATCCCCATAAT^BGCGGCTGTTTTCTG
5hmC Reverse Primer	CAGTTGAGGATCCCCATAATGhmCGGCTGTTTTCTG
156 bp Forward Primer	AACAACTGTTTCAGCCACTGCTTC
75 bp Forward Primer	GCAGCCGGACGTGAACGCGCAG

Supplementary Table 1. Oligonucleotide sequences Sequences used for PCR of synthetic DNA constructs using Lambda DNA as a template. T^B signifies biotinylated thymine.

Sample Label (dsDNA)	Fig.	Pore Diameter (nm)	Measurement Duration (s)
156 bp monobiotinylated DNA (385 nM)	1a&d	7.8	200
156 bp 5hmC labeled DNA (550 nM)	1c-d	8.5	200
Monovalent streptavidin (2.5 µM)	S1	8.5	520
75 bp quantification standard	3a	8.3	320
Genomic DNA NP1 (475 nM)	3b	9.3	512
Genomic DNA NP2 (1 µM)	3b	9.1	640
Genomic DNA NP3 (475 nM)	S7	8.6	806

Supplementary Table 2. SS-nanopore dimensions and measurement times List of nanopore diameters, as determined by ionic resistance, and total time duration of measured traces for each data point in the presented measurements.

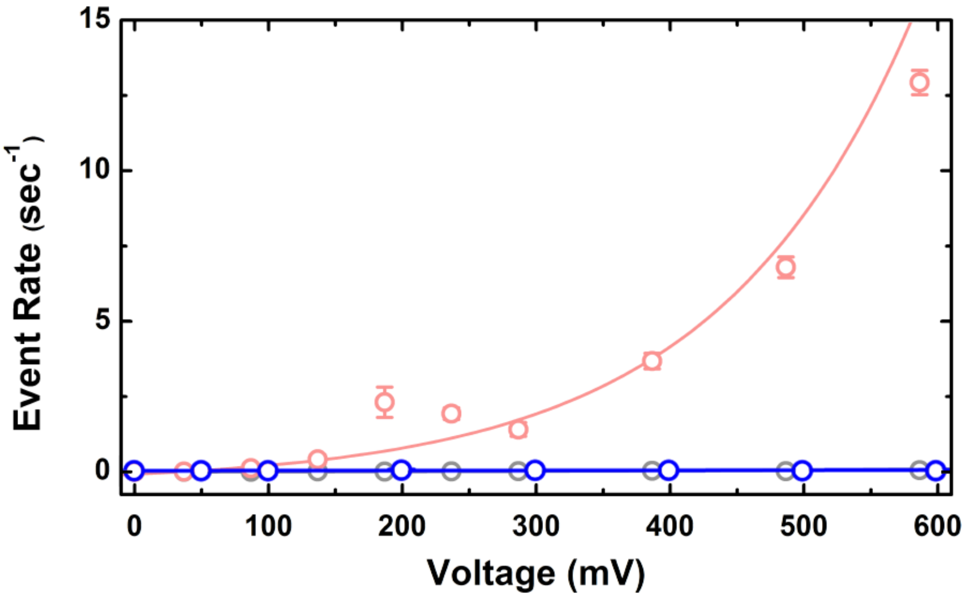

Supplementary Figure 1. MS event rate SS-nanopore event rate for monovalent streptavidin alone (blue, 2.5 μM) measured with a 8.5 nm diameter pore. The background displays rates for biotin-labeled 156 bp mono-5hmC dsDNA (550 nM) both with (red) and without (gray) bound MS for comparison, identical to Fig. 1c from the main text. Solid lines are exponential fits to the data.

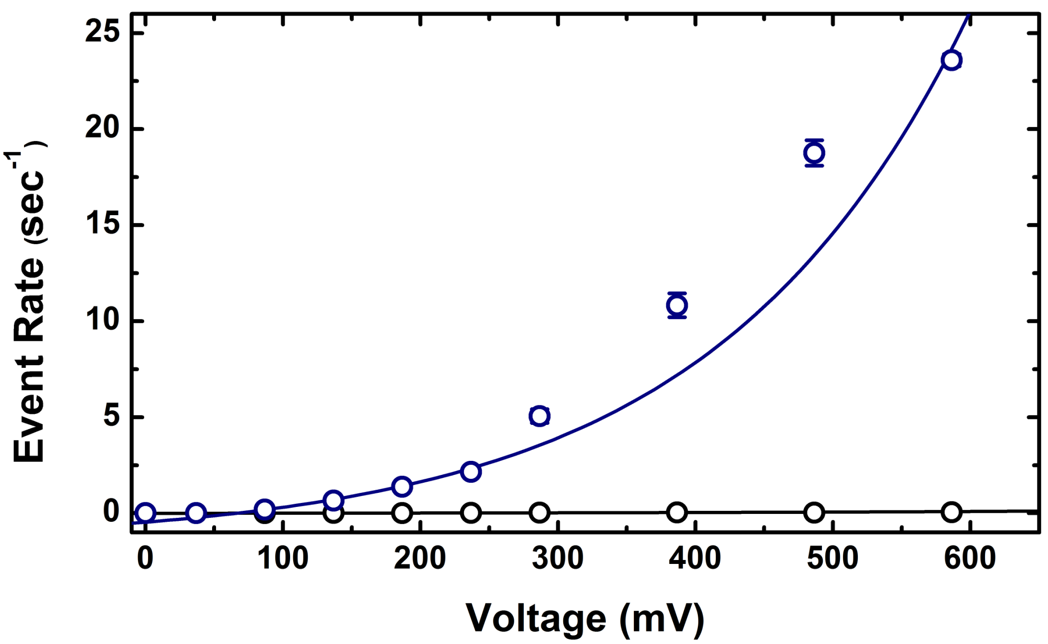

Supplementary Figure 2. Synthetic biotinylated dsDNA event rate SS-nanopore event rates for synthetic 156 bp monobiotinylated dsDNA both with (blue) and without (black) bound MS. DNA concentration for both data sets are 385 nM, similar to the biotin-labeled 5hmC oligonucleotide in Fig. 1c from the main text, considering labeling yield.

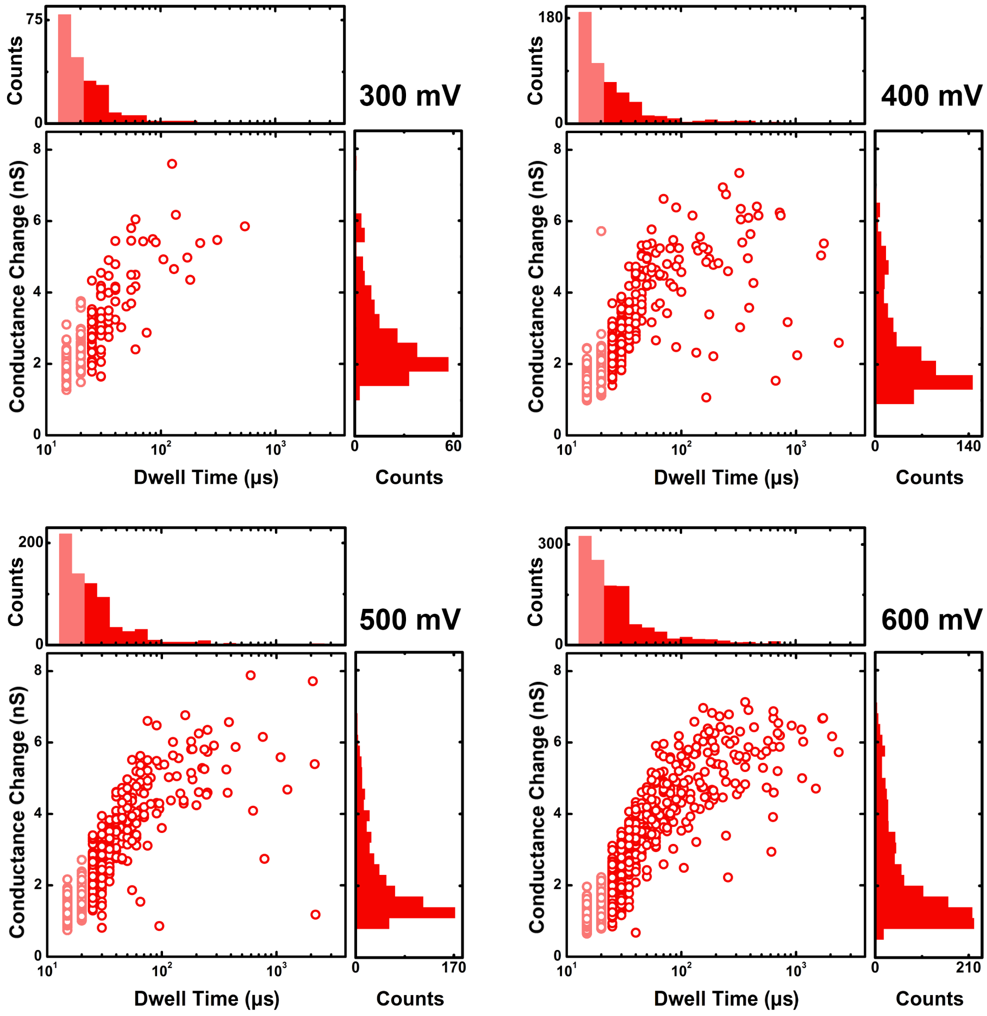

Supplementary Figure 3. Biotin-labeled mono-5hmC scatter plots Mean dwell time vs. mean conductance change scatter plots for 156 bp mono-5hmC dsDNA translocation events (see Fig. 1c from main text) following biotin labeling and incubation with MS. Total number of events considered from 300-600 mV are 217, 529, 717, and 1219, respectively. Faded regions represent events below the resolution limit.

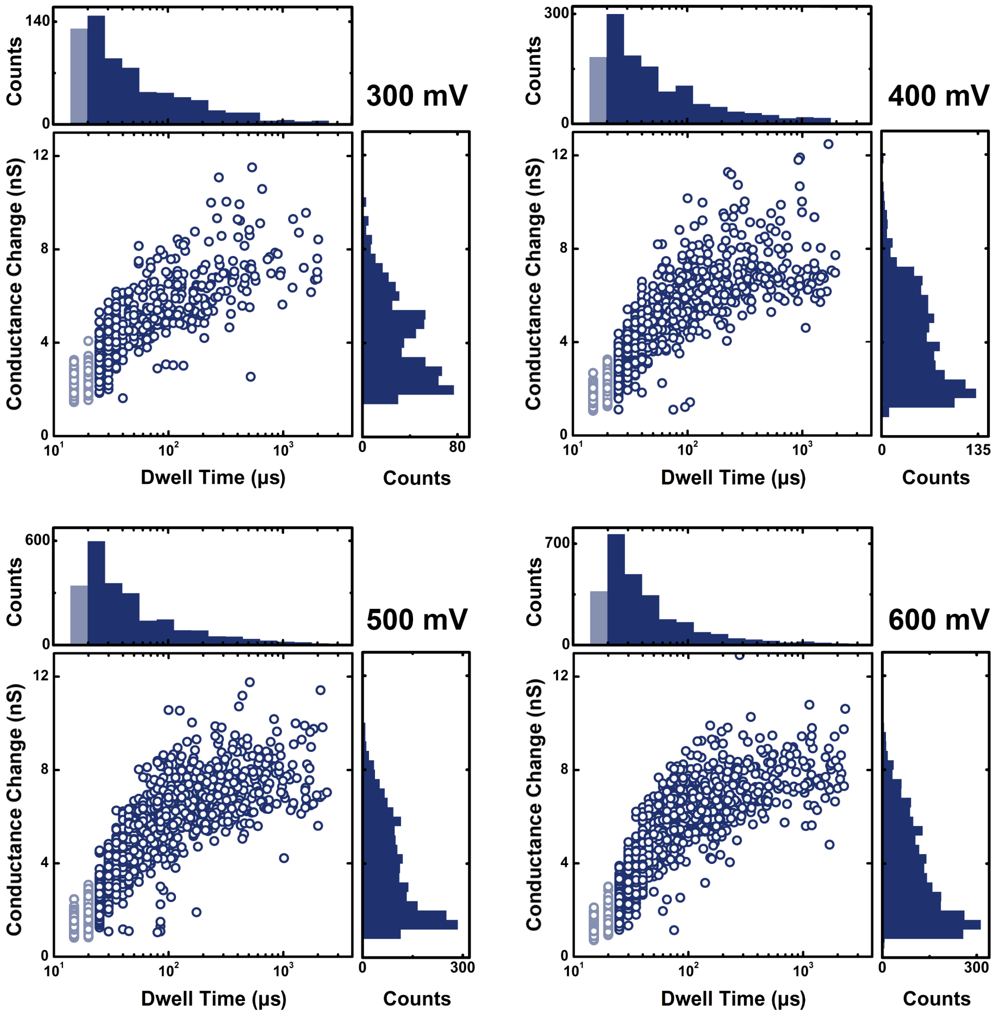

Supplementary Figure 4. Monobiotinylated dsDNA scatter plots Mean dwell time vs. mean conductance change scatter plots for 156 bp synthetic monobiotinylated dsDNA translocation events (see Fig. S1) following incubation with MS. Total number of events considered from 300-600 mV are 673, 1250, 2250, and 2650, respectively. Faded regions represent events below the resolution limit.

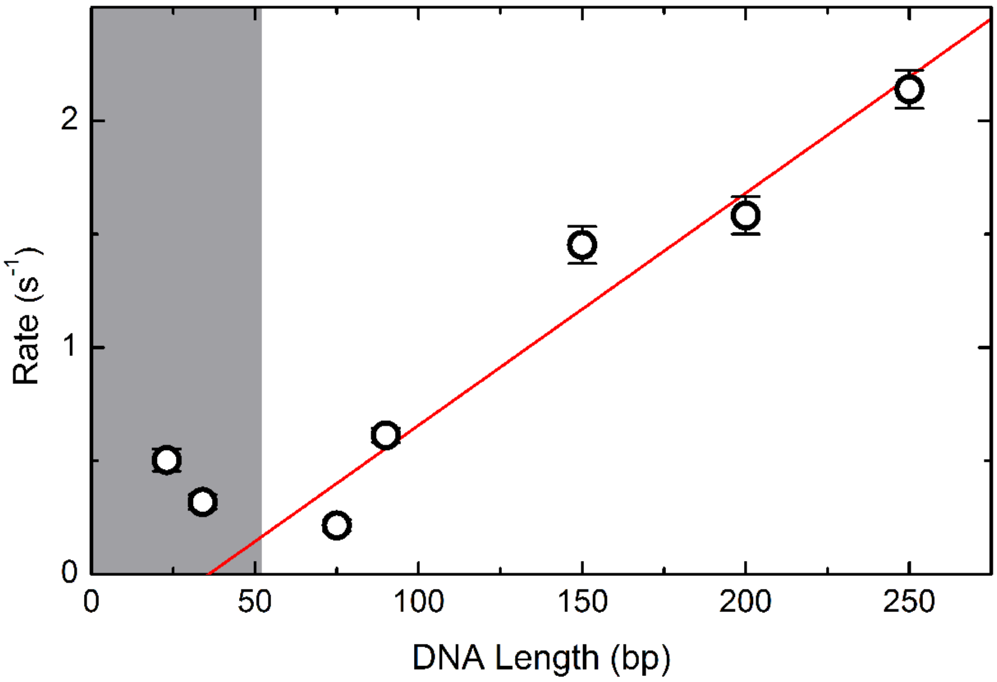

Supplementary Figure 5. Event rate dependence of dsDNA length Translocation event rate (200 mV) for synthetic monobiotinylated dsDNA constructs (1 μM) with bound MS as a function of length. Solid line is a linear fit to data from 75 to 250 bp. Below about 50 bp (shaded region), an unexplained increase in rate is observed. This is the subject of further study but does not impact the present study, which focuses on constructs above this range. dsDNA without bound MS did not show any significant rate across the entire investigated range of lengths.

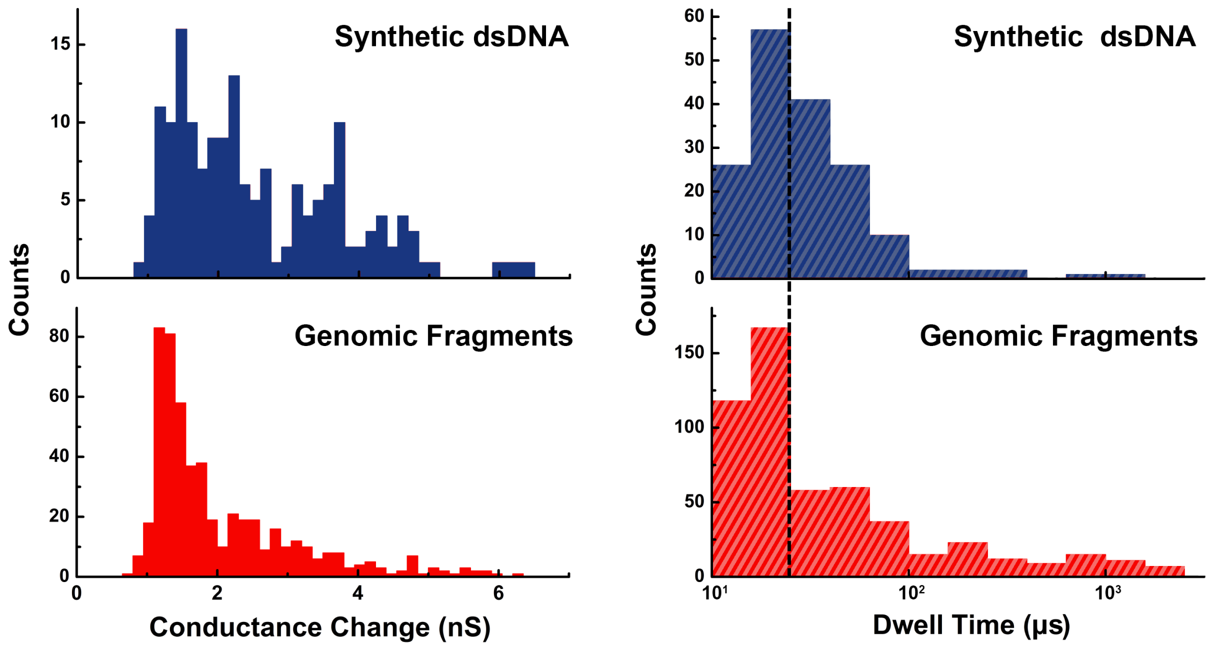

Supplementary Figure 6. Event characteristics of genomic fragments and synthetic dsDNA Mean conductance change (left) and dwell time (right) of translocation events collected at 400 mV for 75 bp synthetic monobiotinylated dsDNA (blue, 50 nM, *n*= 168) and mouse genomic DNA fragmented to an average length of 75 bp and biotin labeled at 5hmC sites (red, c_o= 475 nM, *n*= 492). A qualitatively similar distribution is observed for both materials. Dashed line on dwell time plots represents the resolution limit.

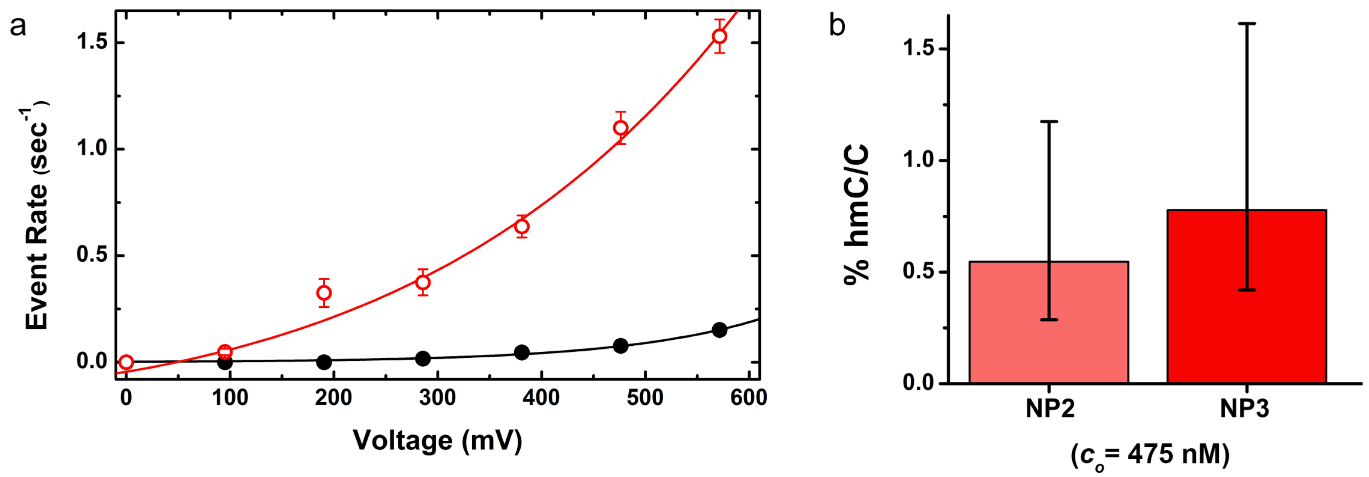

Supplementary Figure 7. Additional 5hmC/C quantification (a) Event rate vs. applied voltage (400 mV) for fragmented genomic DNA (c_o= 475 nM) biotin-labeled at 5hmC sites measured on a third SS-nanopore (NP3, see Table S2). Data shows measurement with (red) and without (black) bound MS and solid lines are exponential fits to the data. (b) Comparison of resulting 5hmC/C ratio R with that of NP2 from the main text (Fig. 3b), also measured at c_o= 475 nM. Quantitative agreement is observed.
